# Validity of the Finnish Diabetes Risk Score for Detecting Undiagnosed Type 2 Diabetes among General Medical Outpatients in Botswana

**DOI:** 10.1155/2016/4968350

**Published:** 2016-09-22

**Authors:** Bernard Omech, Julius Chacha Mwita, Jose-Gaby Tshikuka, Billy Tsima, Oathokwa Nkomazna, Kennedy Amone-P'Olak

**Affiliations:** ^1^Department of Internal Medicine, University of Botswana, Private Bag UB 00713, Gaborone, Botswana; ^2^Department of Public Health, University of Botswana, Private Bag UB 00713, Gaborone, Botswana; ^3^Department of Family Medicine, University of Botswana, Private Bag UB 00713, Gaborone, Botswana; ^4^Department of Ophthalmology, University of Botswana, Private Bag UB 00713, Gaborone, Botswana; ^5^Department of Psychology, University of Botswana, Private Bag UB 00713, Gaborone, Botswana

## Abstract

This was a cross-sectional study designed to assess the validity of the Finnish Diabetes Risk Score for detecting undiagnosed type 2 diabetes among general medical outpatients in Botswana. Participants aged ≥20 years without previously diagnosed diabetes were screened by (1) an 8-item Finnish diabetes risk assessment questionnaire and (2) Haemoglobin A1c test. Data from 291 participants were analyzed (74.2% were females). The mean age of the participants was 50.1 (SD = ±11) years, and the prevalence of undiagnosed diabetes was 42 (14.4%) with no significant differences between the gender (20% versus 12.5%, *P* = 0.26). The area under curve for detecting undiagnosed diabetes was 0.63 (95% CI 0.55–0.72) for the total population, 0.65 (95% CI: 0.56–0.75) for women, and 0.67 (95% CI: 0.52–0.83) for men. The optimal cut-off point for detecting undiagnosed diabetes was 17 (sensitivity = 48% and specificity = 73%) for the total population, 17 (sensitivity = 56% and specificity = 66%) for females, and 13 (sensitivity = 53% and specificity = 77%) for males. The positive predictive value and negative predictive value were 20% and 89.5%, respectively. The findings indicate that the Finnish questionnaire was only modestly effective in predicting undiagnosed diabetes among outpatients in Botswana.

## 1. Introduction

Type 2 diabetes mellitus (T2D) is a common chronic disease globally, and its long-term sequelae include microvascular (retinopathy, nephropathy, and neuropathy) and macrovascular (stroke and myocardial infarctions) complications [1]. Worldwide, T2D accounts for over 90% of cases of diabetes and results from a confluence of environmental, behavioural, and/or genetic factors [2]. Low-income countries, including those in Sub-Saharan Africa (SSA) are projected to have the largest proportional increase in the burden of T2D among adults compared to developed countries by the year 2030 [3]. Most cross-sectional studies in SSA report higher burden of T2D in the urban communities compared to rural settings [4, 5]. This may be attributed to the increasing urbanization and socioeconomic development in the region. For majority of people, urban settings may lead to sedentary lifestyles and unhealthy diets and related obesity, hypertension, dyslipidaemia, and consequently T2D [6]. However, in both communities in SSA, the current decline in communicable diseases like HIV/AIDS, tuberculosis, and malaria has been associated with an increase in life expectancy in the general population leading to the rise of T2D epidemic [7]. Yet, over 50% of cases may be unaware or undiagnosed in SSA including Botswana due to underresourced healthcare systems often resulting in late diagnosis and poor outcomes [8].

T2D is a potentially preventable disease. The majority of cases progress over nearly a decade of asymptomatic phases of prediabetes during which clinically identifiable risk factors are apparent [9]. Although, the benefits of early detection of undiagnosed T2D have not been proven, the potential benefits include appropriate selection of antihypertensive medications [10] and more aggressive risk management to reduce microvascular complications [11]. Currently there are no specific recommendations for diabetes screening in Sub-Saharan Africa (SSA). However, World Health Organization (WHO) recommends organized country-specific opportunistic screening at health facilities or targeted communities [12]. In a resource-poor settings, selective multistage screening is being encouraged by WHO [12]. People regarded to be at high risk are identified after applying preselection criteria. These criteria require population-specific validated diabetes risk score followed by fasting blood glucose and further by oral glucose tolerance test (OGTT) or Haemoglobin A1c (HbA1c) test.

Diabetes risk score provide a cheaper and convenient alternative to mass screening using laboratory based diagnostic tests, which are usually not cost-effective. However, most risk scores were derived and validated for specific Caucasian populations which may not have the same discriminatory accuracy for African populations due to differences in population-specific characteristics [13–15]. Among the existing risk scores, the Finnish Diabetes Risk Score (FINDRISC) is the most valid and inexpensive tool preferred for resource-limited settings by International Diabetes Federation (IDF) [16]. FINDRISC was derived in a 10-year prospective study for identification of people at high risk of future occurrence of T2D among the Finnish population [17]. Follow-up validation in a cross-sectional study to detect prevalence of undiagnosed diabetes showed the area under receiver operating curve (AUROC) of 0.72 in men and 0.73 in women, which is considered a good diagnostic accuracy [18].

Botswana is experiencing a growing prevalence of noncommunicable diseases including T2D in the general population [19, 20]. Validation of a low-cost risk scoring tool such as FINDRISC may enhance public health interventions in targeted populations. The aim of this study was to assess the validity of FINDRISC questionnaire in detecting undiagnosed diabetes among outpatient attendants in Botswana. The diagnostic test used was HbA1c test classified according to the 2015 American Diabetes Association (ADA) criteria [21].

## 2. Materials and Methods

### 2.1. Study Design and Participants

A cross-sectional evaluation was carried out from August to October 2014. The study involved patients aged ≥20 years attending outpatient clinics at Princess Marina Hospital in Gaborone and a district hospital, Letsholathebe II Memorial in Maun, both in Botswana and serviced by a common diagnostic laboratory. We excluded patients who had any acute illness less than two weeks prior to the clinic review and those with previously diagnosed diabetes, documented anaemia, pregnancy, and chronic kidney disease which could interfere with the HbA1c testing accuracy.

### 2.2. Recruitment and Procedure

The study was conducted simultaneously at both clinics. All recruitments and evaluations were done by research nurses who had prior training on the study protocol and good clinical practice certification including anthropometric measures. An average daily attendance of each clinic was about 30 patients. We used a systematic random sampling to recruit five patients per day from each clinic. On each study day, a sampling frame was made from a list of patients on scheduled list. The first participant was randomly picked from the list and thereafter every 6th patient was picked from the list of the patients until sample size for the day was attained. After enrolment, all the patients were evaluated. The sample size was calculated using a sampling error of 5% and power of 80% with anticipated urban prevalence of diabetes of 22.3% [19] and sensitivity of the screening tool of 78% [17]. This resulted into an estimated sample size of 266 participants, which was increased by 5% (*n* = 279 participants) to account for possible dropouts. Informed consents were obtained for all the participants who met the eligibility criteria.

After obtaining informed consent, the following data were collected from each participant: FINDRISC risk parameters, demographic characteristics, hyperglycaemic symptoms, history of chronic diseases (including hypertension), and human immunodeficiency virus (HIV) infection, blood pressure, and anthropometric measurements. FINDRISC score form is a one-page questionnaire containing eight simple questions of risk factors for T2D that does not require any invasive procedures. The risk categories include age (years), body mass index (kg/m^2^), waist circumference (cm), daily consumption of fruits, berries, or vegetables, daily physical activity (having at least 30 minutes of physical activity during work or at leisure time), history of antihypertensive drug treatment, history of high blood glucose, and family history of diabetes mellitus in the first-degree or second-degree relatives. Every category is assigned with weighted scores according to its associated risk, and the final scores range from 0 to 26 points [18]. Each participant is classified according to their future risk of developing type 2 diabetes as follows; “low risk” if total score is <7 points, “slightly elevated risk” if total score is 7–14 points, “moderate risk” if total score is 12–14 points, “high risk” if total score is 15–20 points, and “very high risk” if total score is >20 points.

The weight was measured to the nearest 0.1 kg and the standing height was measured to the nearest 0.1 cm by using a stadiometer attached to the same medical balance weighing scale (*HI-CARE Int*,* India*). The body mass index (BMI) was calculated as weight per square meters (kg/m^2^). Waist circumference (WC) was taken by a nonstretchable tape measure at a level midway between the lowest rib and iliac crest to the nearest 0.1 cm as described in the original study [17].

Venous blood samples for HbA1c were obtained from all the participants and analyzed in one laboratory using a standardized high-performance liquid chromatography (HPLC) assay method [*Abbott Architect*,* 2007*,* Germany*]. The method is aligned with the Diabetes Control and Complications Trial (DCCT) research groups and certified by National Glycated Haemoglobin Standardization Program (NGSP) [22]. The HbA1c results were categorized according to American Diabetes Association criteria into normal glycaemia (<5.6%), dysglycaemia (5.7–6.4%), and diabetes (≥6.5%) [21]. All participants with undiagnosed diabetes were referred to the physician for case management. The study was approved by the ethical committees of the two hospitals, University of Botswana and Ministry of Health, and carried out in compliance with Helsinki Declaration.

### 2.3. Statistical Analysis

Statistical analyses were conducted using SPSS for Windows (*version 23.0*;* SPSS Inc.*,* Chicago*,* IL*,* USA*). Descriptive data were expressed as means (± standard deviation) for continuous variables and proportions for categorical variables. Comparisons of the differences between genders were carried out by Student's *t*-test for continuous variables and the Chi-squared test for categorical variables. The diagnostic accuracy of FINDRISC to detect undiagnosed diabetes was evaluated using the area under receiver operating characteristic (AUROC) curve, and the sensitivity (the proportion of true positive results) and specificity (the proportion of true negative results) were estimated by nonparametric method. The sensitivity was plotted against *y*-axis and false positive rates (1 − specificity) against *x*-axis. An area under the curve (AUC) of 0.5 indicated that the test performed is no better than chance and AUC of 1.0 indicated perfect discrimination. The optimal cut-off points were determined by the point with the closest distance to (0, 1) in the ROC curve which maximizes the sensitivity and specificity of the test. To obtain optimal cut-off point, we calculated this distance for each observed cut-off point and located the point where the distance is minimum for (1 − sensitivity)^2^ + (1 − specificity)^2^ [23]. The positive and negative predictive values were estimated. All statistical tests were two-sided and a *P* value of less than 0.05 was considered statistically significant.

## 3. Results

### 3.1. Participants' Characteristics

A total of 704 outpatient attendees were selected. Of these, 304 (43.2%) patients were excluded because of documented evidences of being acutely ill, known diabetes status, chronic kidney disease, anaemia, or refusal to consent. In addition, 109 (15.5%) participants were excluded due to incomplete data. Of the 291 participants enrolled, 216 (74.2%) were females and all were residing in the urban centres of Gaborone and Maun. Mean age of the study population was 50.1 (SD = ±11) years. The most common chronic comorbidities were hypertension (40.2%) and HIV infection (39.2%). Mean BMI, waist circumference and total FINDRISC scores were higher in women than in men (Table 1). Females had significantly higher prevalence of overweight (31.5% versus 18.7%, *P* < 0.01), obesity (41.7% versus 17.3%, *P* < 0.01), and abdominal obesity (65.3% versus 18.7%, *P* < 0.01) compared to males (Table 1).

**Figure 1:**
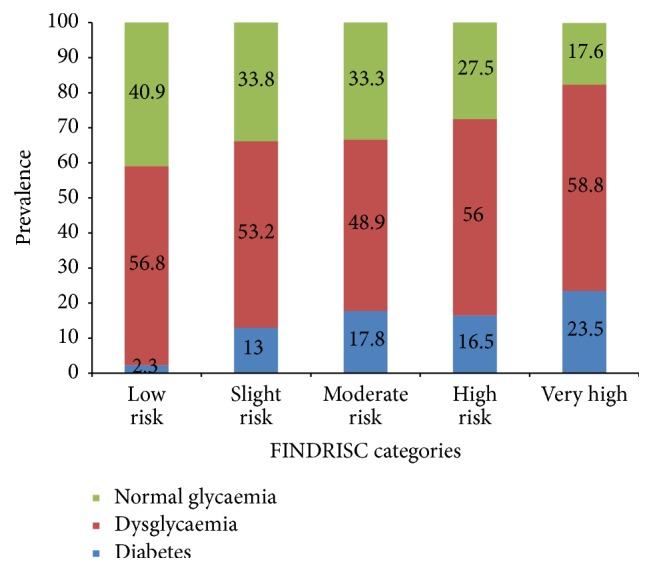
The distribution of normal glycaemia, dysglycaemia, and undiagnosed T2D within the FINDRISC categories among the participants. The figures are percentage prevalence within the FINDRISC category.

The overall prevalence of undiagnosed diabetes was 42 (14.4%) and there was no significant difference between women and men (20% versus 12.5%, *P* = 0.26). The prevalence of undiagnosed diabetes increased progressively with the increase in FINDRISC scores. The total prevalence of dysglycaemia was 54.6% and the corresponding prevalence within the risk groups was uniformly distributed among the FINDRISC categories (see Figure 1).

**Figure 2:**
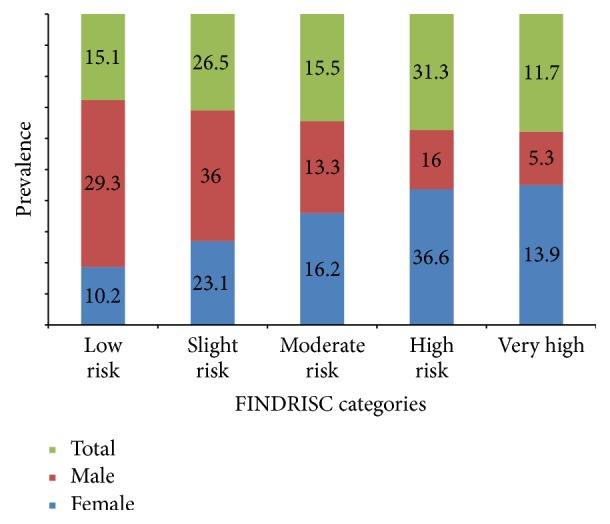
Distribution of FINDRISC categories by gender. The figures are percentage prevalence of each risk category by gender compared to the total prevalence in each category. Females had significantly higher prevalence of high risk categories than men (*P*-value < 0.001).

In total, 49.5% of the participants had low-to-moderate risk of T2D and 50.5% had high or very high risk to develop T2D in the next 10 years according to the FINDRISC scale (see Figure 2). Out of the 42 (14.4%) individuals with undiagnosed diabetes, 55% were in the high and very high risk categories. Women had significantly higher prevalence of high risk categories than men (*P* < 0.001). The most prevalent risk factors for T2D in these populations were inadequate intake of fruits and vegetables (86.9%), physical inactivity (61.9%), hypertension (46.9%), and obesity (41.7% in women, 17.3% in men).

### 3.2. Diagnostic Accuracy of FINDRISC

**Figure 3:**
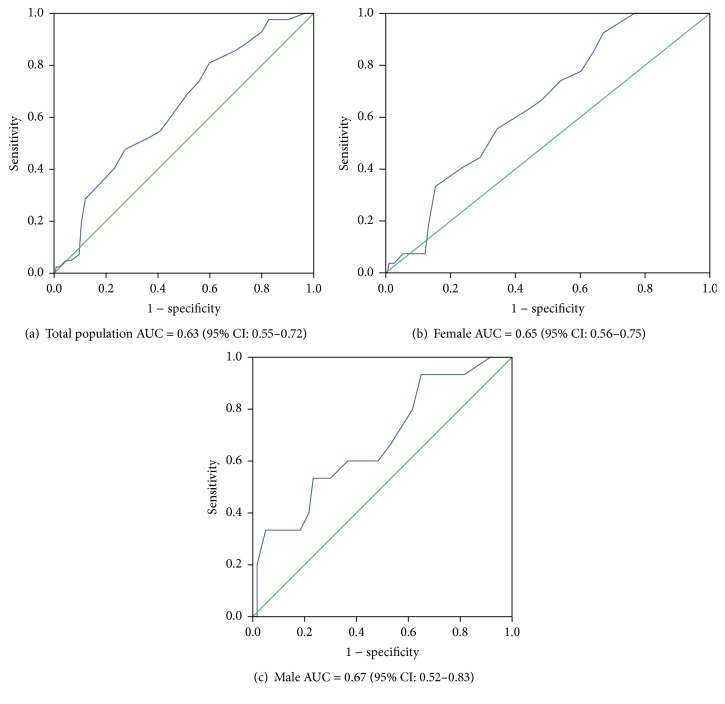
The receiver operating characteristic (ROC) curves for detecting undiagnosed T2D using FINDRISC and HbA1c (%) among outpatients in Botswana: (a) total population, (b) females, and (c) males. (a–c) Diagonal segments are produced by ties.

The AUROC was 0.63 (95% CI: 0.55–0.72) for the total population, 0.67 (95% CI: 0.52–0.83) for men, and 0.65 (95% CI: 0.56–0.75) for women (see Figure 3). The optimal cut-off point was at 17 for the overall population, with sensitivity and specificity of 48% and 73%, respectively. For females, the optimal cut-off was the same as the overall population but the sensitivity and specificity were at 56% and 66%, respectively. Men had a lower optimal cut-off of 13 with sensitivity and specificity of 53% and 77%, respectively. The total positive predictive value (PPV) at cut-off value of 17 points was 20% and corresponding negative predictive value (NPV) was 89.7%.

## 4. Discussion

FINDRISC has been widely adopted as a low-cost screening tool in many European countries to enable early identification of individuals at risk of T2D who might benefit from early preventive interventions [18, 24]. Although it is widely recommended for use in low-resource settings [16], it has not been validated in SSA populations [13]. The sensitivity and specificity of FINDRISC at optimal score were 56% and 66% for women and 53% and 77% for men, respectively. The area under curve (AUC) value was 0.67 for women and 0.65 for men. This performance was lower than in the first cross-sectional validation in the Finnish population [18] but was not different from a large primary healthcare study in Spain which found AUC of 0.67 using the same diagnostic test [25]. Previous validation studies using OGTT as the diagnostic test, however, still showed diminished performance of FINDRISC in different populations with AUC ranging between 0.67 and 0.75 [24, 26, 27]. While there are limited data on prior use of FINDRISC in African countries, a simplified FINDRISC model (using 6 questions and omitting diet and physical activity) in a mixed racial community in Cape Town used OGTT as the diagnostic test and found comparable AUC [28].

Although the overall accuracy of the risk score in this study is sufficiently effective based on AUC [29], its overall sensitivity appears low (48%) at optimal cut-off value of 17 based on the ROC curve estimation. However, when each gender is considered separately, the sensitivity was greater, suggesting that the small number of male participants in the study could have contributed to poor overall sensitivity. Apart from the differences in population characteristic, the modest performance of the risk score in this population may be attributed to the use of HbA1c. OGTT is still the gold standard test for undiagnosed diabetes in high risk groups [25, 30]; though HbA1c has very high specificity in different racial groups, it has a lower sensitivity [31, 32]. The use of HBA1c requires no fasting and was previously reserved for mostly monitoring blood glucose control over the previous 3-month duration for known cases of diabetes, but it has since been approved as a confirmatory diagnostic test [33]. However, the test is not validated in some countries, including Botswana.

Undiagnosed T2D was present in 14.4% of our study population. Even though this is not nationally representative sample, our findings suggest a high burden of the disease in this group. According to IDF estimates, more than half of diabetes cases in Africa are undiagnosed [8]. In Botswana, the national prevalence of T2D is estimated at about 4%–8% with a wide disparities between the rural and urban communities [19, 20]. PPVs of predictive models are dependent on diseases prevalent; we found a PPV of 20% which is greater than similar studies on prevalence of undiagnosed T2D [24, 25]. Finding of a correspondingly high NPV in our study is also reassuring because people who test negative can be correctly ruled out [34]. Currently, there is no screening strategy for undiagnosed T2D in Botswana; in the absence of any perfect screening method, always a trade-off between simplicity and accuracy is needed. Thus, FINDRISC is a simple and considerably valid questionnaire in this population as a preliminary screening method that can be followed by more invasive and accurate diagnostic test for high risk individuals.

Most current guidelines on screening for asymptomatic adults for T2D do not recommend routine screening for adults considered to have low-to-moderate risks. For adults at high risk, the recommendation is to screen with fasting plasma glucose every 3 to 5 years, with annual screening for those deemed to be at very high risk [35, 36]. The prevalence of modifiable risk factors including obesity and hypertension for T2D among the participants was very high, which are potential targets for preventive interventions. Such interventions may include lifestyles modifications for those identified to be at high risk [37].

The primary limitation to this study was the use of HbA1c test, a nonvalidated diagnostic test for T2D in this country that may have contributed to lower performance. Therefore, a T2D screening strategy based solely on FINDRISC in this population may run a risk of giving false reassurance to a significant proportion of individuals who are considered low risk. Secondly, it should also be noted that FINDRISC does not include other known T2D risk factors such as cardiovascular diseases and other insulin resistance states which require diagnostic testing regardless of the score. Thus, future studies in the same population using OGTT are needed to validate our findings. Thirdly, demographics of the populations were skewed towards females, which could have significantly underpowered the performance of the risk score in the male gender, hence leading to imprecise estimates.

In conclusion, although, FINDRISC's performance was only modestly effective in detecting undiagnosed T2D in this population, its overall accuracy is not so different from similar studies in other populations. It can still play a useful role in diabetes screening strategy in Botswana in outpatient clinic setting due to its simplicity and moderate accuracy. Further studies based on OGTT are needed in the general population to determine its predictive capacity in SSA population.

## Figures and Tables

**Table 1:** General characteristics of the participants (*N* = 291).

Participants	Total	Male	Female	*P* value
Age (years ± SD)	50.1 (11)	50.5 (11.8)	49.9 (11.0)	0.68
Body mass index (kg/m^2^ ± SD)	28.0 (6.5)	24.6 (5.5)	29.2 (6.5)	<0.001
Waist circumference (%)	72.7	34.7	85.7	<0.01
BP systolic (mmHg ± SD)	128.6 (19.3)	130 (21.1)	128 (18.6)	0.51
BP diastolic (mmHg ± SD)	78.5 (12.1)	78.6 (11.8)	78.4 (12.1)	0.9
Total FINDRISC (points ± SD)	13.2 (5.9)	10.1 (5.2)	14.3 (5.8)	<0.001
HbA1c (% ± SD)	5.9 (0.76)	6.0 (0.6)	5.9 (0.8)	0.63

All figures are in means except waist circumference which is proportion (females > 80 cm and males > 94 cm).

SD = standard deviation. BP = blood pressure.
